# Relationship between periodontitis, type 2 diabetes mellitus and COVID-19 disease: a narrative review

**DOI:** 10.3389/fcimb.2025.1527217

**Published:** 2025-05-08

**Authors:** José Luis Muñoz-Carrillo, Paulo Israel Palomeque-Molina, Marcelo Stalin Villacis-Valencia, Oscar Gutiérrez-Coronado, Francisca Chávez-Ruvalcaba, Silverio Jafet Vázquez-Alcaraz, Paola Trinidad Villalobos-Gutiérrez, Josue Palomeque-Molina

**Affiliations:** ^1^ Laboratorio de Inmunología, Centro Universitario de los Lagos, Universidad de Guadalajara, Lagos de Moreno, Jalisco, Mexico; ^2^ Escuela de Odontología, Global University, Aguascalientes, Aguascalientes, Mexico; ^3^ Facultad de Ciencias de La Salud, Escuela de Odontología, Universidad Internacional del Ecuador, Quito, Ecuador; ^4^ Licenciatura en Nutrición, Universidad Autónoma de Zacatecas, Zacatecas, Zacatecas, Mexico; ^5^ Departamento de Odontología Conservadora, Escuela de Odontología, Universidad Complutense de Madrid, Madrid, Spain; ^6^ Departamento de Pediatría, Hospital Homero Castanier Crespo, Azogues, Ecuador

**Keywords:** COVID-19, inflammation, periodontitis, SARS-CoV-2, type 2 diabetes mellitus

## Abstract

Inflammation plays a fundamental role in the development and bidirectional association of di-verse diseases, such as periodontitis and type 2 diabetes mellitus (T2DM), which generates important clinical complications, where chronic exposure to high levels of blood glucose affects the repair process of periodontal tissues. Likewise, it has been observed that comorbidity, between these two diseases, influences the development of the COVID-19 disease towards a more severe course. However, there is currently very little scientific evidence on the relationship between periodontitis, T2DM and COVID-19 disease. This narrative review aims to provide an understanding of the current and most relevant aspects of the relationship between periodontitis, T2DM and COVID-19 disease. A narrative review was performed through a systematic search of published studies, without date restrictions, indexed in the electronic databases of PubMed, for the inclusion of articles in English, and LILACS for the inclusion of articles in Spanish. This review included different articles, which addressed the most important aspects to present a current perspective on the relationship and influence between periodontitis, T2DM and COVID-19 disease. Comorbidity between periodontitis and T2DM represents a greater risk of developing a more severe course of COVID-19 disease, because these three diseases share three important axes: a clinicopathological axis; an axis associated with glycemia, and an immunological axis associated with inflammation.

## 1 Introduction

At a clinical level, periodontal health is characterized by the absence of inflammation (Chapple et al., 2018). Under these conditions, the periodontal tissue is capable of adequately defending itself, through various mechanisms of the immune system, against the presence of bacteria present in the oral cavity. Periodontal disease develops when the balance between these defense mechanisms that control infection and the subgingival biofilm is lost, triggering the innate (inflammation) and adaptive immune response of the host (Mira et al., 2017). Periodontal disease can be divided into four stages based on the type of lesion: 1) initial and 2) early lesions; which are part of gingivitis; and the 3) established and 4) advanced lesions that are part of periodontitis (Muñoz-Carrillo et al., 2019). In this context, periodontitis is an immunoinflammatory disease that mainly affects the periodontal tissues that support the teeth, causing their progressive destruction, which ultimately results in tooth loss (Kedlaya et al., 2023). On the other hand, there are risk factors that influence the development and severity of periodontal disease, which can be local and systemic. Likewise, these factors can also be modifiable, such as smoking, stress, obesity, and uncontrolled diabetes mellitus, among others; and non-modifiable such as sex, age, ethnicity or genetic factors (Moreno Caicedo et al., 2018).

Diabetes mellitus is a syndrome that involves a wide variety of genetic, epigenetic and pathophysiological abnormalities, which can be influenced by environmental factors, such as infections, diet (nutrients), intestinal microbiota, among others (Hanson and Godfrey, 2015; Stančáková and Laakso, 2016; Sircana et al., 2018; Zhang and Pollin, 2018). T2DM is the most common type of diabetes. T2DM is characterized by presenting various defects at a biochemical and pathophysiological level, which are associated with peripheral insulin resistance, increased hepatic glucose production, altered levels of intestinal hormones that regulate insulin and glucagon function, decrease and failure of pancreatic β cells function, as well as additional mechanisms that are related to inflammation (Defronzo, 2009; Brunton, 2016; Javeed and Matveyenko, 2018). In the context of T2DM, insulin plays an essential role in the regulation of immunocellular function, acting as a critical link between metabolic dysfunction and the immune response (Berbudi et al., 2025). Insulin resistance, a central feature of T2DM, disrupts immunological homeostasis by affecting the functionality of innate and adaptive immune cells, resulting in an imbalance between pro-inflammatory and anti-inflammatory responses (Dror et al., 2017). This state favors the overproduction of proinflammatory cytokines and adipokines, including tumor necrosis factor alpha (TNF)-α, interleukin (IL)-6, leptin, and resistin, which not only exacerbate insulin resistance but also contribute to the establishment of a chronic low-grade inflammatory microenvironment (Gerrits et al., 2012; Villarreal-Pérez et al., 2014; Sun et al., 2018). This persistent inflammation constitutes a key pathogenic factor in the progression of T2DM, further impairing insulin signaling and compromising carbohydrate metabolism (Dror et al., 2017).

Several studies have associated T2DM with periodontitis, suggesting a bidirectional association between both pathologies (Santos et al., 2015; Turner, 2022), since patients with T2DM have a greater probability of developing periodontitis, and in those patients who present this comorbidity, between both pathologies, they show worse blood glucose control (Tsai et al., 2002; Guzman et al., 2003; Liccardo et al., 2019). In this context, T2DM leads to an increase in the expression of proinflammatory cytokines in periodontal tissues (Polak and Shapira, 2018), such as IL-1β and prostaglandin (PG)-E_2_ in gingival crevicular fluid. Likewise, an increase in the expression of TNF-α, IL-1β, IL-6, IL-17, and IL-23 in the gingiva has been reported, both in patients and in animal models with diabetes (Salvi et al., 1997; Salvi et al., 1998), which influences the vascular and cellular phenomena of inflammation (Domingueti et al., 2016), stimulating greater bone resorption, through an increase and reduction in the expression of the receptor activator of nuclear factor-κ B ligand (RANKL) and osteoprotegerin, respectively (Santos et al., 2010). On the other hand, inflammation also induces an increase in the production and activation of matrix metalloproteinases, which leads to the destruction of connective tissue, induction of apoptosis in fibroblasts and osteoblasts, thus limiting the repair capacity of the periodontal tissues (Pacios et al., 2013; Sarkar et al., 2013; Xiao et al., 2016). Furthermore, a decrease in the production of anti-inflammatory lipid mediators and cytokines such as IL-4, IL-10 and transforming growth factor (TGF)-β has been reported, potentially contributing to the development and aggravation of periodontal inflammation in patients with T2DM (Andriankaja et al., 2012; Acharya et al., 2017; Van Dyke, 2017).

Another important factor during T2DM is the role of blood glucose concentration, since high blood glucose levels contribute to the development and evolution of inflammation, through the activation of various intracellular signaling pathways. For example, mitogen-activated protein kinase (MAPK) and nuclear factor (NF)-κB pathways, which results in an increase in the production of proinflammatory mediators, such as cytokines and reactive oxygen species (Coughlan and Sharma, 2016; Fakhruddin et al., 2017; Lim et al., 2017; Zheng et al., 2018). Furthermore, it has been observed that patients with T2DM show an increase in both the expression of inducible nitric oxide synthase (iNOS) and the levels of lipid peroxides in the periodontium and crevicular fluid, respectively, which contributes to a more severe course of the periodontal inflammation (Shaker et al., 2013).

The COVID-19 disease, caused by SARS-CoV-2, has caused alarming numbers of infections and deaths around the world (Zhang et al., 2023). The clinical characteristics of the COVID-19 disease are very diverse, which can present from an asymptomatic state, or mild symptoms can manifest (Ullah et al., 2021); until progressing to pneumonia, developing acute respiratory distress syndrome (ARDS), multiple organ dysfunction and death (Ye et al., 2020). The pathophysiology of COVID-19 disease may not be limited exclusively to pulmonary manifestations, including pneumonia and ARDS (Gupta et al., 2020), since SARS-CoV-2 is able to infect other cell types which express its binding receptor, angiotensin-converting enzyme (ACE)-2 (Muniyappa and Gubbi, 2020), such as cells of the upper respiratory system, alveolar epithelial cells in lungs, enterocytes, endothelial cells (Gurwitz, 2020), from heart (Zheng et al., 2020), tubular epithelium kidney (Diao et al., 2021) and pancreas (Liu et al., 2020), causing organ-specific extrapulmonary clinical manifestations associated with harmful effects on many other systems of the human body, such as neurological, thrombotic, endocrine, cardiac, dermatological, hepatic, renal and gastrointestinal (Gupta et al., 2020). Although it is known that the majority of people with COVID-19 do not develop symptoms or only have mild manifestations of the disease, approximately 14% of infected people develop the disease with a severe course (Zhou et al., 2020), where advanced age and some comorbidities, such as diabetes (Abdi et al., 2020), have been associated as potential risk factors for triggering more severe disease and death (Zhou et al., 2020). Diabetic patients who suffer from COVID-19 have a prevalence of death between 22 to 31%, compared to patients without diabetes (Singh et al., 2020). Elements that could influence in patients with diabetes mellitus to increase susceptibility to COVID-19 disease include: greater ease for the virus to adhere and efficiently enter cells, less effectiveness of the immune system in eliminating the virus, greater probability of suffering severe complications due to the excessive release of proinflammatory cytokines causing hyperinflammation, and presence of diseases associated with the heart (Muniyappa and Gubbi, 2020). Likewise, it has been shown that there is high expression of ACE-2 in the lung, kidney, heart, and pancreas in rodent models of diabetes mellitus (Wysocki et al., 2006; Roca-Ho et al., 2017), and a higher pulmonary expression of ACE-2 in humans (Rao et al., 2020). In this context, diverse studies support the hypothesis that patients with diabetes mellitus have greater susceptibility to SARS-CoV-2 infection, since they are not able to efficiently eliminate the virus. This is due, on the one hand, to the fact that patients with diabetes mellitus have high levels of furin, a protease involved in cleaving the S1 and S2 domains of the virus spike protein, which facilitates the entry of the virus into the cell (Fernandez et al., 2018). Furthermore, patients with diabetes mellitus present alterations in the immune system, which inhibit neutrophil chemotaxis, phagocytosis, and intracellular destruction of pathogens, as well as delaying both the activation of Th1 cells and the hyperinflammatory response (Hodgson et al., 2015; Cuschieri and Grech, 2020). On the other hand, patients with COVID-19 present, at a peripheral level, low counts of CD4+ and CD8+ T cells, but with a higher proportion of pro-inflammatory CD4+ Th17 T cells, along with high levels of pro-inflammatory cytokines (Yang et al., 2020).

Currently, there is not enough scientific evidence on the relationship between periodontitis and T2DM and the risk of SARS-CoV-2 infection. Therefore, a more extensive and exhaustive search is necessary to identify additional literature; and in this way provide a more reliable and accurate hypothesis and conclusion about the association of these three pathologies. In this context, the aim of this research was to provide a systematized narrative review to contrast the existing evidence on the relationship between periodontitis, T2DM and COVID-19 disease. In this narrative review, a systematic methodology was applied (Page et al., 2021), without date restrictions, indexed in the electronic databases of PubMed, for the inclusion of articles in English, and LILACS for the inclusion of articles in Spanish, through the use of the Boolean operators AND, OR and NOT; using the following DeCS/MeSH terms: “periodontal disease”, “periodontitis”, “type 2 diabetes mellitus”, “SARS-CoV-2” and “COVID-19”.

## 2 Periodontitis and type 2 diabetes mellitus

**Figure 1:**
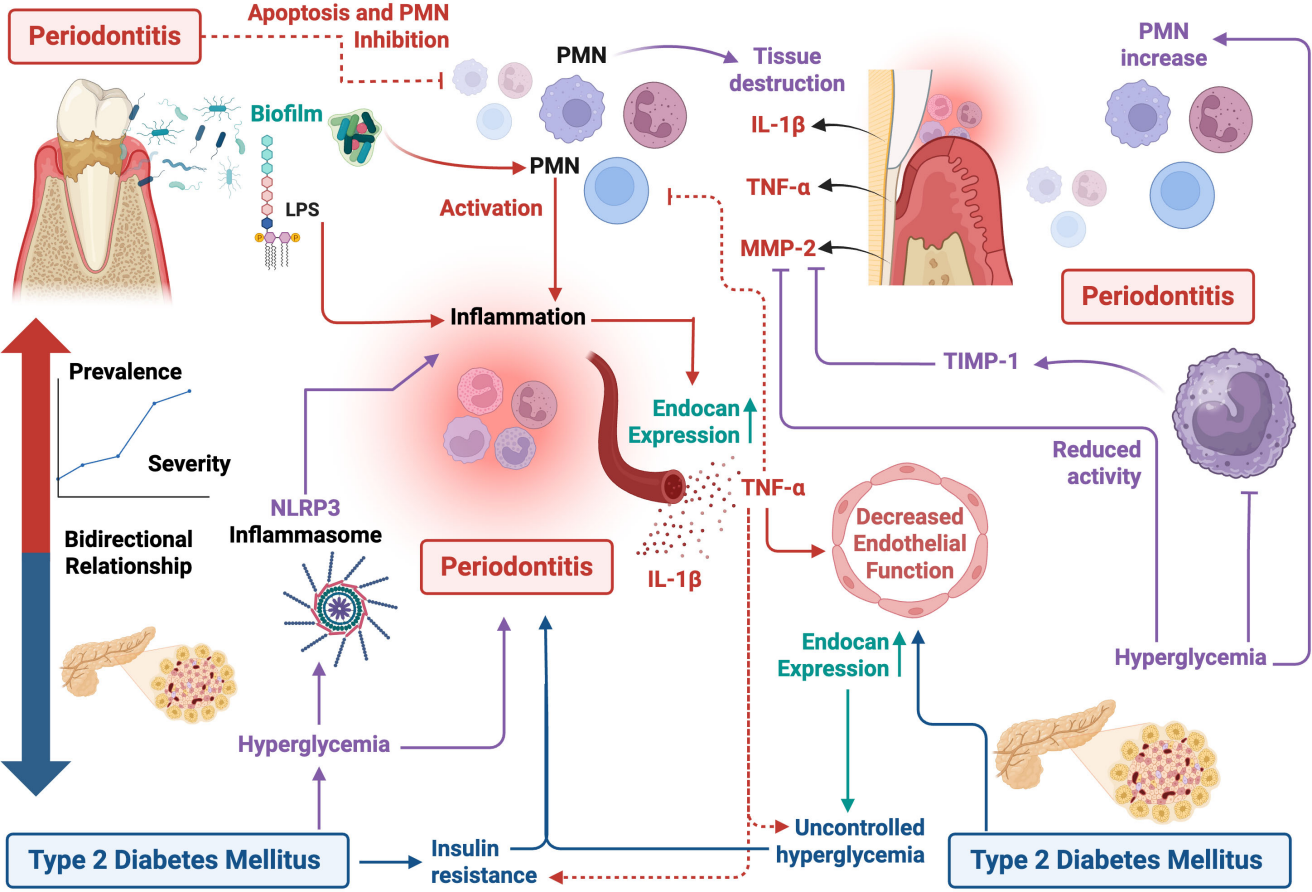
Pathogenic inflammatory mechanisms between periodontitis and type 2 diabetes mellitus. The figure represents the bidirectional interaction between both pathologies, in which periodontal inflammation contributes to a systemic proinflammatory state, characterized by an increase in the release of proinflammatory cytokines (such as TNF-α and IL-1β), which induce insulin resistance and worsen glycemic control. In parallel, chronic hyperglycemia typical of T2DM generates oral dysbiosis, immune dysfunction, endothelial dysfunction, and increased susceptibility to periodontal disease progression. This inflammatory and immunometabolic responses establishes a pathogenetic cycle that enhances the severity of both conditions. Figure created with BioRender.com by Muñoz-Carrillo et al.

Periodontitis is considered the sixth complication of diabetes mellitus (Acharya et al., 2017), because several studies have shown a strong bidirectional relationship between these diseases, since it has been observed that in subjects with T2DM (controlled or not) present a significant increase in the prevalence of chronic or severe periodontitis, compared to healthy subjects (Susanto et al., 2011; Shamala et al., 2017; Alasqah et al., 2018; Trentin et al., 2018; Wu et al., 2020; Monod Nuñez et al., 2022). This bidirectional relationship between periodontitis and T2DM is also because both diseases share pathogenic inflammatory mechanisms (**Figure 1**). On the one hand, periodontitis can influence the development and state of chronic systemic inflammation, through the aberrant increase in proinflammatory cytokines, such as IL-1β, IL-6 and TNF-α, affecting endothelial function, and substantially contributing to the development of insulin resistance, causing a homeostatic imbalance in blood glucose regulation (Acharya et al., 2017). On the other hand, T2DM is closely related to vascular endothelial dysfunction, affecting the protective balance and permeability of the endothelium, enhancing chronic systemic inflammation (Janket et al., 2008; Gurav, 2014; Li et al., 2022).

TNF-α plays a crucial role in regulating the expression of endocan, a soluble proteoglycan that is highly produced by vascular endothelium during endothelial activation and inflammatory processes (Sarrazin et al., 2006). This dual characteristic allows that the endocan may act as both an inflammatory mediator and a marker of endothelial activation (van Eijk et al., 2014). Interestingly, studies have shown a correlation between elevated endocan levels and worsening glycemic control; while improvements in glycemic control lead to a decrease in endocan expression. Furthermore, endocan expression has been detected in systemically healthy individuals with periodontal disease (Arman et al., 2016; Türer et al., 2017), suggesting a potential link beyond glycemic status. In this context, endocan could be a promising biomarker for the early diagnosis and prognosis of chronic inflammatory states in T2DM and periodontal disease, due to its ability to reflect the impact of endothelial activation in these pathological conditions. Furthermore, endocan could serve as an indicator for monitoring the response to treatment in patients with T2DM and periodontal disease, since the alteration of its levels is associated with the inflammatory state and glycemic control in individuals with these pathologies (Kumar et al., 2020).

On the other hand, a hallmark of metabolic disorders, particularly T2DM, is the abnormal activation of both the innate and adaptive immune systems, through the recruitment of immune cells in the affected tissues, even in the absence of external pathogens or antigens (Coelho et al., 2013; Zhou et al., 2018). The direct consequences of these responses and the modulation of immune cell populations depend largely on the metabolic system, altering cellular functionality, increasing the secretion of cytokines and chemokines, as well as the recruitment and activation of leukocyte populations (Lackey and Olefsky, 2016). Therefore, the hyperglycemia in patients with T2DM favors the increase of polymorphonuclear neutrophil leukocytes (PMNs) within the tissues, altering several functions such as cell adhesion, chemotaxis, phagocytosis, and the degradation of antigens, generating tissue damage by these cells. Because periodontitis and T2DM share a complex relationship involving inflammation, hyperinflammation, especially caused by hyperreactive PMNs, plays a crucial role in host tissue destruction in the pathogenesis of periodontitis, since the different phenotypes that present by PMNs act as an important link in both diseases, influencing in their pathogenesis (**Figure 1**) (Ling et al., 2015).

Bacteria residing in the gingival sulcus trigger the activation of PMNs, resulting in an increase in the release of molecules with bactericidal properties. These molecules, in turn, are considered responsible for the hallmark characteristics that mark the progression of periodontal disease, including the destruction of periodontal (Nussbaum and Shapira, 2011) tissue and inflammation, which may contribute to metabolic dysregulation (Ling et al., 2015). In this context, Herrmann et al. found that patients with periodontitis and T2DM showed a significant increase in gingival PMNs, compared to individuals who only had periodontitis, indicating a hyperinflammatory reaction in the gingival tissue, probably due to T2DM. Therefore, it is suggested that inflammation may be a bilateral factor that can increase the severity and progression of both diseases (Herrmann et al., 2020). The research by Manosudprasit et al. corroborates these findings. In their study, it was observed that the apoptosis of PMNs in the peripheral blood was altered in individuals with T2DM. Furthermore, periodontal disease acted as a confounding factor, meaning that it exerted an additive effect, significantly delaying spontaneous PMNs apoptosis in patients with T2DM and periodontitis. These findings suggest that periodontal disease not only affects the apoptosis of PMNs at the site of periodontal infection but also has a systemic impact on the resolution of inflammation and clearance of PMNs. This may contribute to the exacerbation of other systemic inflammatory conditions, such as T2DM. In fact, it has been shown that apoptosis of PMNs is delayed in periodontal disease due to the action of TNF-α (**Figure 1**) (Manosudprasit et al., 2017).

Furthermore, T2DM is considered a significant risk factor for the development of periodontitis (Dhir et al., 2018), because T2DM intensifies the inflammatory response in periodontal tissues, significantly increasing the levels of proinflammatory mediators such as IL-1β and TNF-α, as well as an increase in the activity of matrix metalloproteinases (MMP) (Mesia et al., 2016). On the other hand, high blood glucose levels attenuate the immune response in patients with T2DM, affecting the recovery of periodontal tissue, which alters the etiopathology of diverse diseases, such as periodontitis (Grover and Luthra, 2013). MMPs are enzymes that play a crucial role in tissue remodeling and the breakdown of the extracellular matrix (ECM) (Sapna et al., 2014). Furthermore, they are involved in the regulation of the activity of various biologically active substrates (Collazos et al., 2015), such as pro- and anti-inflammatory cytokines, chemokines, growth factors, serum components, complement components and cell signaling molecules, which modulate immune responses (Heikkinen et al., 2016). MMP-2 is a highly active MMP present in saliva, which plays a crucial role in the degradation of periodontal tissues (Woessner, 1991). Recent studies have established a connection between MMP-2 and periodontitis, since its activity is controlled by tissue inhibitors of matrix metalloproteinases (TIMPs) (Bătăiosu et al., 2015), mainly TIMP-1, a natural inhibitor of MMP-2 produced by periodontal cells, macrophages and monocytes (**Figure 1**) (da Costa Fernandes and Zambuzzi, 2020).

During periodontal tissue inflammation, an overexpression of MMP-2 has been observed in saliva and gingival crevicular fluid (Lira-Junior et al., 2017; Barreiros et al., 2018). In the study carried out by Arreguin-Cano et al. (2019), the periodontal status, HbA1c levels, MMP-2 and TIMP-1 activity, and percentage of PMNs in patients with T2DM were compared and analyzed. In this study, an increase in the enzymatic activity of MMP-2 was observed, as well as the expression of TMP-1 according to the severity of periodontitis, this increase being significant in severe periodontitis. In addition, a significant increase in glycosylated hemoglobin (HbA1c) levels was found in patients with moderate and severe periodontitis, suggesting that poor glycemic control is associated with the severity of periodontitis. Likewise, it was observed that in patients with poor glycemic control, there was a significant increase in PMNs, along with a significant decrease in MMP-2 and TMP-1 activity. These findings suggest that in patients with T2DM and poor glycemic control there is an imbalance in MMP-2/TIMP-1, and that the process of inhibition of MMP-2 activity by TIMP-1 is lost in severe periodontitis (**Figure 1**) (Arreguin-Cano et al., 2019).

On the other hand, the NLRP3 inflammasome plays an important role during the inflammatory response against infections or cellular stress (Schroder and Tschopp, 2010). In this context, studies have reported a high expression of the NLRP3 inflammasome, both in the gingival tissues of patients with periodontitis (Bostanci et al., 2009; Park et al., 2014), as well as in cells of the innate immune system and pancreatic β-cells in patients with T2DM (Schroder et al., 2010; Jourdan et al., 2013). In this context, Huang et al. (2015) reported that, both in patients with chronic periodontitis and T2DM, as well as human gingival epithelial cells (HGEC) stimulated with lipopolysaccharide (LPS) and high concentrations of glucose, showed a significant increase in the expression of the NLRP3 inflammasome and IL-1β. These findings suggest that hyperglycemia can exacerbate the inflammatory response of gingival tissue through the NLRP3 pathway, contributing to greater tissue degradation (Huang et al., 2015), because high levels of IL-1β were significantly associated with periodontitis immunopathology, causing periodontal tissue degradation, mainly in alveolar bone absorption and damage to the lamina propria (**Figure 1**) (Liu et al., 1996).

## 3 Periodontitis and COVID-19

**Figure 2:**
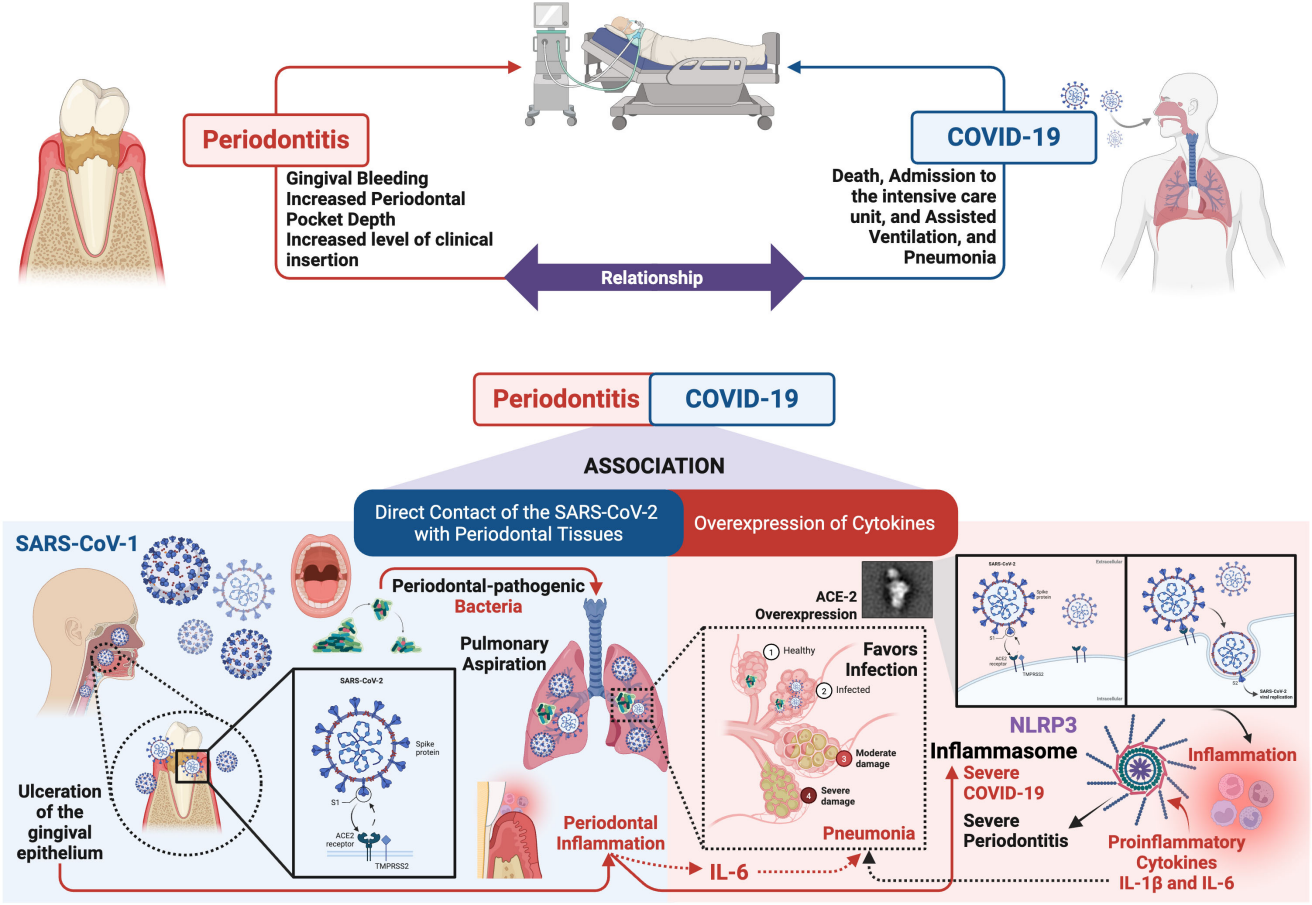
Relationship between periodontitis and COVID-19. The figure illustrates the potential immunopathological mechanisms linking periodontal disease with SARS-CoV-2 infection. Periodontitis induces a chronic systemic inflammatory state characterized by the overproduction of proinflammatory cytokines (such as IL-6), which may contribute to an exacerbated immune response in COVID-19 patients, favoring the development of a “cytokine storm.” Additionally, the oral dysbiosis associated with periodontitis may facilitate opportunistic colonization by respiratory pathogens, increasing susceptibility to severe pulmonary infections. Furthermore, it has been suggested that periodontal inflammation may upregulate the expression of receptors such as ACE-2, potentially enhancing viral entry. This evidence supports the hypothesis that periodontal status could influence the clinical severity of COVID-19. Figure created with BioRender.com by Muñoz-Carrillo et al.

At a clinical level, studies have established an association between periodontitis and adverse outcomes of COVID-19 (Brock et al., 2022). Patients with periodontal disease have been shown to be at increased risk of severe COVID-19, including hospitalization, intensive care unit admission, and mortality. Furthermore, periodontal disease may contribute to the severity of COVID-19 by elevating levels of inflammatory biomarkers (Marouf et al., 2021; Gupta et al., 2022). Conversely, COVID-19 can exacerbate periodontal disease, leading to increased gingival bleeding, dental plaque accumulation, and periodontal pocket deepening (**Figure 2**) (Anand et al., 2022; Kalsi et al., 2022). Although there is currently no clear causal relationship, periodontitis represents a risk factor for increasing the severity of COVID-19 (Sánchez Sánchez et al., 2022), by causing microbial dysbiosis, bacterial super-infection, hyperreactivity of the host, and over stimulation of the immune system. Probably due to the set of environmental, microbial and inflammatory factors, which contribute to the progression of the disease (Sukumar and Tadepalli, 2021). According to Campisi et al. (2021) two interrelated mechanisms may underlie the association between periodontitis and COVID-19. The first mechanism involves a direct viral infection of periodontal tissues, facilitated by the high expression of the ACE-2 receptor in these tissues. The second mechanism involves a shared inflammatory response (overexpression of inflammatory cytokines) characterized by a cytokine storm, a condition associated with severe COVID-19 (Campisi et al., 2021) (**Figure 2**).

*Direct contact of the SARS-CoV-2 with periodontal tissues.* Periodontitis-induced ulceration of the gingival epithelium may compromise its protective function, increasing the risk of SARS-CoV-2 invasion. The main route of entry of SARS-CoV-2 into human cells is through the ACE-2 receptor, present in diverse tissues such as the lungs, nasal passages, salivary glands, and oral cavity (Huang et al., 2021). In the mouth, ACE-2 is mainly found in tongue cells, fibroblasts, periodontal tissues, and gingival crevices (Mancini et al., 2020). Notably, the salivary glands of the oral cavity have higher expression of ACE-2 than the lungs, making them an important reservoir of the virus and facilitating effective infection (Jiménez et al., 2022). In addition to ACE-2, other molecules such as TMPRSS-2, and furin, are required for SARS-CoV-2 infection (Sakaguchi et al., 2020). These molecules are highly expressed in the oral cavity, especially in the oral lining, gingival cells, periodontal tissue, and gingival fluid (Pascolo et al., 2020). Their combined presence is crucial for the activation of the the S protein of SARS-CoV-2, allowing it to bind to host cells and enhance its ability to infect the oral cavity (Campisi et al., 2021; Sukumar and Tadepalli, 2021). In this context, periodontal-pathogenic bacteria, such as *Porphyromonas gingivalis*, can induce the overexpression of ACE-2, TMPRSS2, and furin in cells of the oral cavity (Sena et al., 2021). This overexpression of ACE-2, on the one hand, negatively regulates the production of proinflammatory cytokines, such as IL-1β, IL-6, and TNF-α (Mancini et al., 2020); while on the other hand, it favors the entry of SARS-CoV-2 into the oral cavity (Brock et al., 2022). Once SARS-CoV-2 infection occurs, ACE-2 expression is downregulated, leading to an increase in proinflammatory cytokines, favoring the inflammatory response (Iwasaki et al., 2020). In this sense, local inflammation promotes the spread of SARS-CoV-2 infection and its replication in periodontal tissues, with possible further systemic expansion. Therefore, it is deduced that aspiration of periodontal-pathogenic bacteria could increase the risk of SARS-CoV-2 infection, since these can increase the expression of ACE-2 in the oral cavity, lungs, and bronchi; inducing the production of inflammatory cytokines, such as interleukin IL-6, by alveolar and bronchial epithelial cells, which promotes SARS-CoV-2 infection, and inflammation of the lower respiratory tract can become severe in the presence of pneumonia viral, contributing to the development of cytokine storm and acute respiratory distress syndrome (**Figure 2**) (Campisi et al., 2021; Brock et al., 2022).

*Overexpression of cytokines.* IL-6, a cytokine overexpressed in periodontitis, has been implicated in the pathogenesis of COVID-19 (Silvestre and Márquez-Arrico, 2022). SARS-CoV-2 infection induces the release of proinflammatory cytokines, including IL-1β and IL-6, which may contribute to the development of interstitial pneumonia, a hallmark of severe COVID-19. While the causal role of IL-6 in COVID-19 severity remains under investigation, it has been proposed as a potential biomarker for early disease detection and progression monitoring (**Figure 2**) (Campisi et al., 2021). In this context, serum IL-6 levels have been correlated with the stage of COVID-19 disease, particularly in patients experiencing respiratory failure. Therefore, elevated IL-6 levels can be used as a predictive biomarker to identify patients at risk for disease progression. Furthermore, increased expression of the IL-6 receptor (IL-6R) and higher levels of IL-6 have been observed in COVID-19 patients who did not survive compared to patients who survived throughout the clinical course of the disease. These findings suggest a potential role of IL-6 in the pathogenesis and progression of COVID-19 (Qi et al., 2023).

Periodontitis and COVID-19 share several common inflammatory pathways, such as the NLRP3/IL-1β and IL-6 signaling pathway (**Figure 2**). NF-κB induces the transcriptional expression of NLRP3 and pro-IL-1β (Liu et al., 2017; Chen et al., 2021). Activation of the NLRP3 inflammasome results in the release of pro-inflammatory cytokines IL-1β and IL-18 (Brodin, 2021), thereby promoting inflammation and other associated disorders. Inflammatory cytokines can promote the development of low-grade systemic inflammation, leading to the abnormal activation of the NLRP3 inflammasome. This, in turn, can drive chronic inflammatory conditions and influence the pathophysiology of inflammation-related diseases (Sharma and Kanneganti, 2021). It has been observed that patients with periodontitis exhibit significantly higher levels of NLRP3, in both blood and saliva. NLRP3 inflammasome-related proteins, such as IL-1β, have been proposed as potential biomarkers for periodontal clinical status (Qi et al., 2023). Studies have reported that the expression of these proteins is associated with alveolar bone loss, a hallmark of periodontal disease, and an increase in proinflammatory cytokines, which can contribute to the severity of periodontal disease (**Figure 2**) (Mainas et al., 2022). COVID-19 severity has been correlated with NLRP3 inflammasome activation. Post-mortem analysis of COVID-19 patients has revealed persistent NLRP3 inflammasome activation in various tissues and PMNs from peripheral blood (Qi et al., 2023). This is because, after viral replication, ACE-2 decreases its activity, activating ACE1, leading to elevated levels of PMN, reactive oxygen species, NF-κB, and proinflammatory cytokines, ultimately resulting in inflammatory cell death and tissue damage (Ariana Dalys and Fabricio Miltom, 2023).

## 4 Type 2 diabetes mellitus and COVID-19

**Figure 3:**
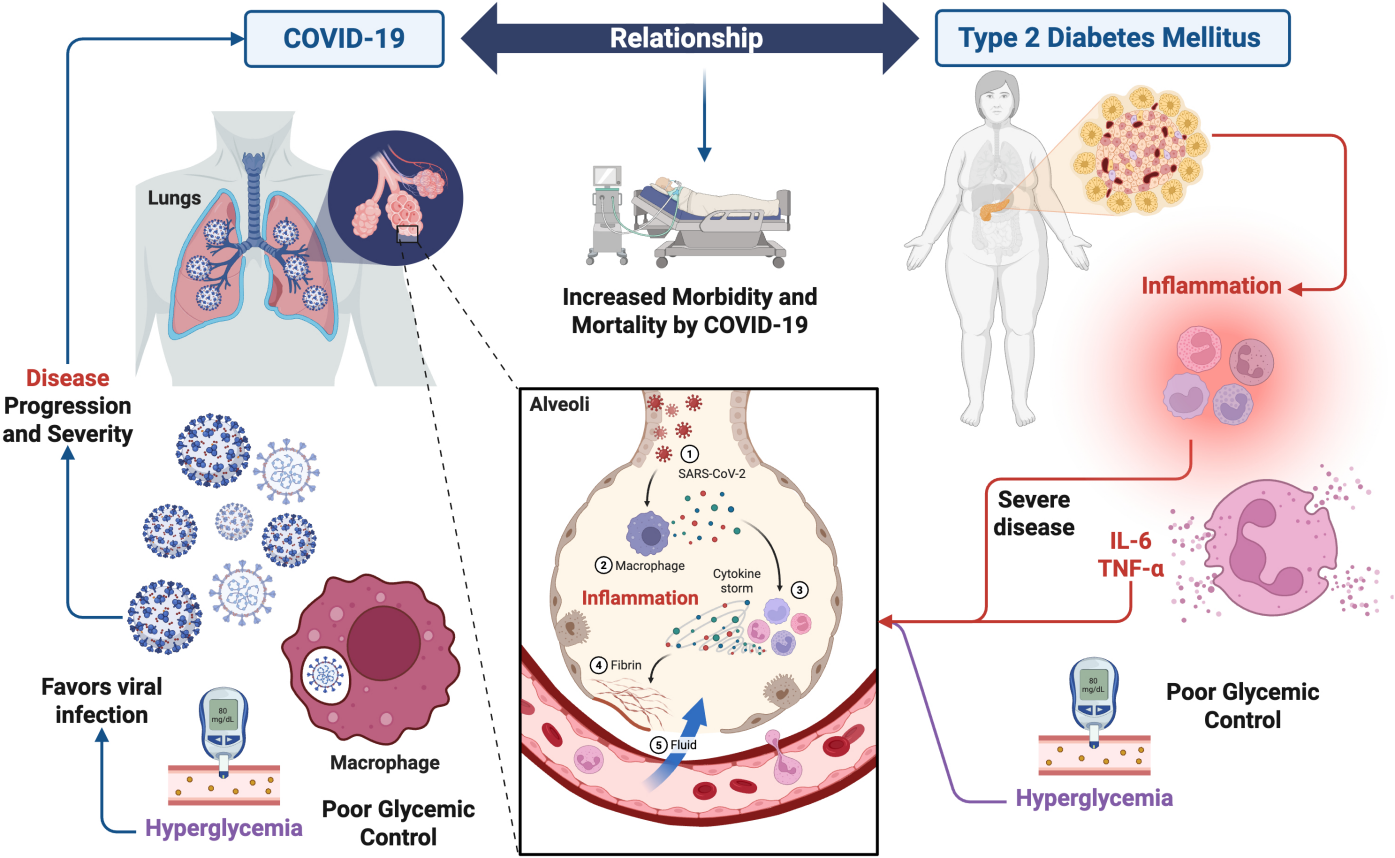
Relationship between type 2 diabetes mellitus and COVID-19. The figure depicts the pathophysiological mechanisms by which T2DM may influence the clinical course of SARS-CoV-2 infection. Chronic hyperglycemia in individuals with diabetes contributes to persistent inflammatory state marked by elevated levels of proinflammatory cytokines such as IL-6, and TNF-α. These factors can amplify the dysregulated immune response characteristic of severe COVID-19, promoting cytokine storm, acute lung injury, and adverse clinical outcomes. This interaction suggests that T2DM is not only a risk factor for severe COVID-19 but also actively contributes to its immunopathological progression. Figure created with BioRender.com by Muñoz-Carrillo et al.

Retrospective studies of this group of patients indicate that poor glycemic control is associated with increased morbidity and mortality from COVID-19. However, the severity of COVID-19 is closely correlated with the age of patients, which is often also the case for T2DM (Turk Wensveen et al., 2021). It has been reported that hospitalized patients with COVID-19 and T2DM have almost double the risk of mortality compared to their counterparts without diabetes (Shenoy et al., 2020). In addition, COVID-19 positive patients with T2DM had worse clinical outcomes, exhibiting a severe inflammatory response with a higher risk of admission to the intensive care unit, receiving mechanical ventilation, and in-hospital mortality than those without diabetes (**Figure 3**) (Shenoy et al., 2020; You et al., 2020).

The underlying molecular mechanism of how T2DM leads to more severe COVID-19 disease is currently unclear (Turk Wensveen et al., 2021). However, this susceptibility of patients with T2DM to adverse outcomes associated with SARS-CoV-2 infection is due to impaired immune system function, and possible up regulation of enzymes that mediate viral invasion. Chronic inflammation caused by diabetes, coupled with the acute inflammatory reaction caused by SARS-CoV-2, results in a propensity for inflammatory storm (**Figure 3**) (Yin et al., 2021); which is characterized by the following successive stages: 1) Infection of lung cells by SARS-CoV-2; 2) immune cells, including macrophages, identify the virus and produce cytokines; 3) cytokines attract more immune cells, such as white blood cells, which in turn produce more cytokines, creating a cycle of inflammation that damages lung cells; 4) damage can occur through fibrin formation; and 5) weakened blood vessels allow fluid to leak and fill the lung cavities, causing respiratory failure (**Figure 3**).

Likewise, it has been reported that patients with T2DM and COVID-19 had a higher count of white blood cells, neutrophils, and proinflammatory cytokines (such as IL-6 and TNF-α), suggesting an increased inflammatory response compared to patients without diabetes (Shenoy et al., 2020). In addition to this, the severity of hyperglycemia was associated with the intensity of the cytokine storm, which is a clear indication that immunological triggers are responsible for changes in blood glucose regulation in the context of a severe disease. Furthermore, a fundamental role of alveolar macrophages has been indicated, which increase their glycolytic rate after activation. In this context, SARS-CoV-2 can infect macrophages and benefit from the increase in the glycolytic rate in these cells. Therefore, the presence of a hyperglycemic state in patients with T2DM further facilitates viral replication in macrophages, promoting disease progression (**Figure 3**) (Turk Wensveen et al., 2021).

## 5 Relationship between periodontitis, type 2 diabetes mellitus and COVID-19

In the current scientific literature, there is only one systematic review, whose purpose was to carry out a systematic review of the literature, which included 12 studies, to contrast the existing evidence on the relationship between periodontal disease and diabetes mellitus, and the risk of SARS-CoV-2 infection, as well as to establish a hypothesis that explains the ways in which this interaction could occur. Casillas Santana et al. (2021) hypothesize that the relationship between these three pathologies is because T2DM is a metabolic disorder characterized by hyperglycemia in the blood, the result of altered secretion or action of insulin. Likewise, periodontitis and diabetes mellitus are inflammatory disorders with a bidirectional association, which share a similar immunomodulatory cascade and cytokine profile. On the other hand, ACE-2 is a crucial component of the renin-angiotensin system, and a key entry factor into SARS-CoV-2 cells. ACE-2 is widely distributed in various tissues including the oral cavity, mainly in the tongue and periodontal tissue. ACE-2 expression is modified by chronic uncontrolled glycemia in T2DM. Therefore, uncontrolled hyperglycemia increases the risk of developing periodontitis and triggers an overexpression of ACE-2 in the periodontal tissue of patients with T2DM, these events being potentially essential for SARS-CoV-2 infection and the development of the mild to severe form of COVID-19 (Casillas Santana et al., 2021). However, this systematic review was carried out in 2021, and has certain limitations, mainly in the search strategy, since the studies evaluated are limited to the English language, excluding studies conducted in Spanish. Therefore, a broader and more exhaustive search is necessary to identify additional literature; and in this way provide a more reliable and precise hypothesis and conclusion on the association of these three pathologies. In this context, this review provides a comprehensive, original and exhaustive perspective on the influence and association of COVID-19 disease with T2DM and periodontitis, through three axes, which interrelate these three pathologies.

**Figure 4:**
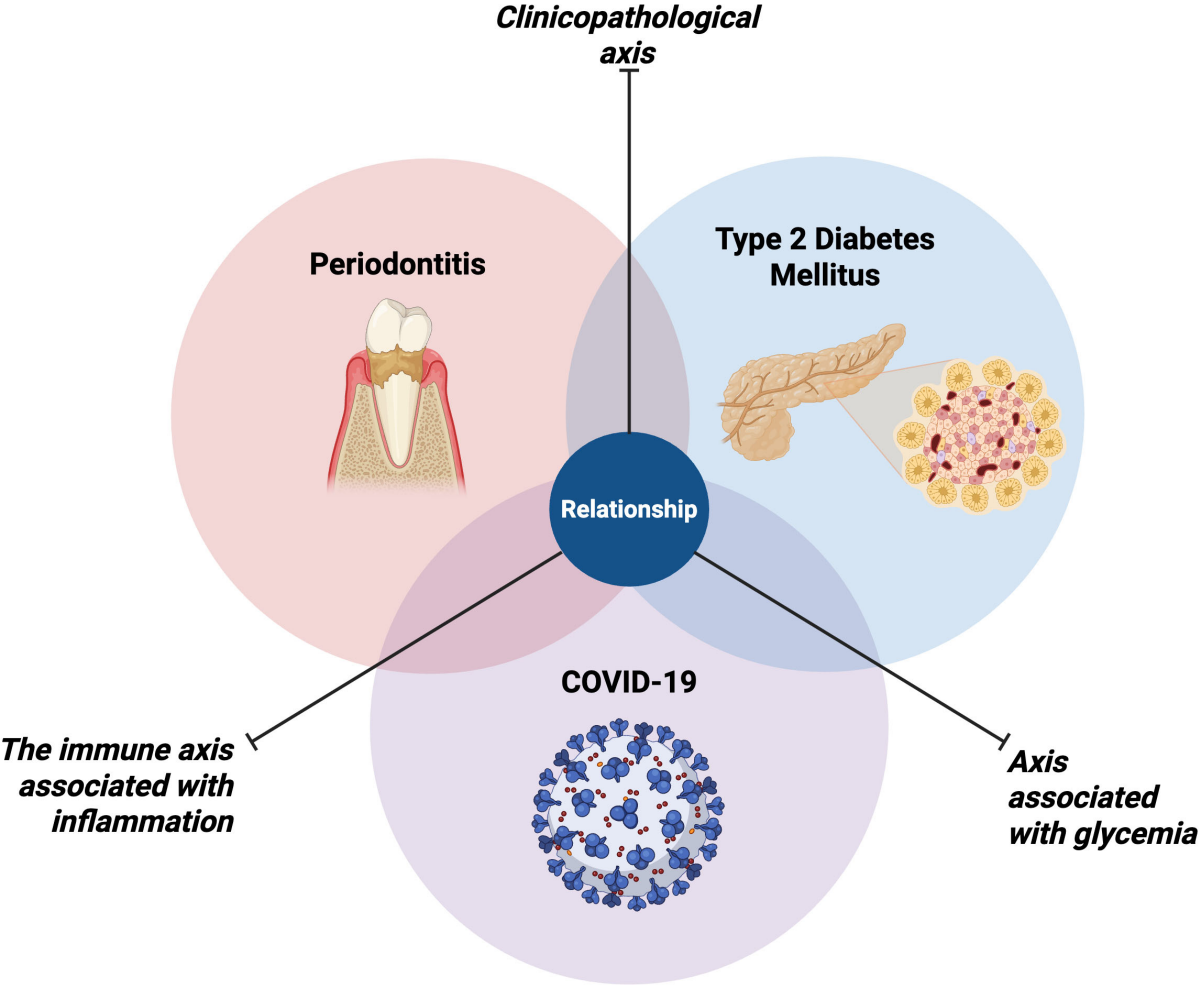
Relationship between periodontitis, type 2 diabetes mellitus, and COVID-19. The figure illustrates the complex interplay between these three chronic conditions, which share three important axes that interconnect them: 1) a clinicopathological axis; 2) an axis associated with glycemia; and 3) an immune axis associated with inflammation. Figure created with BioRender.com by Muñoz-Carrillo et al.

Currently, there is very little scientific literature on the relationship between periodontitis, T2DM, and COVID-19. In the present study, we first sought to explain the relationship between the following comorbidities: 1) periodontitis and T2DM; 2) periodontitis and COVID-19; and 3) T2DM and COVID-19. Based on the systematized reviewed literature, in addition to the few published scientific studies on the relationship between these three pathologies, we can hypothesize that the three diseases share important cofactors (**Table 1**), which focus on three important axes (**Figure 4**): 1) a clinicopathological axis; 2) an axis associated with glycemia; and 3) an immune axis associated with inflammation.

**Table 1:** Interrelated cofactors between periodontitis, type 2 diabetes mellitus, and COVID-19.

Co-factor	Periodontitis	Type 2 Diabetes Mellitus (T2DM)	COVID-19	Ref
Impact on the immune system	Chronic inflammation weakens immune defense.	T2DM leads to immune dysfunction and impaired inflammatory response.	COVID-19 exacerbates immune dysfunction and inflammation.	(Coelho et al., 2013; Mira et al., 2017; Zhou et al., 2018; Berbudi et al., 2025)
Effect on inflammation	Inflammation in periodontal tissues can spread to other parts of the body.	Chronic inflammation from T2DM worsens the response of the body to infections.	COVID-19 triggers systemic inflammation, which can worsen comorbid conditions.	(Moreno Caicedo et al., 2018; Muniyappa and Gubbi, 2020; Turk Wensveen et al., 2021; Yin et al., 2021)
Glycemic control	Poor oral health can negatively affect glycemic control.	Poorly controlled T2DM worsens COVID-19 outcomes due to impaired immune function.	Inflammatory responses from COVID-19 may disrupt glucose metabolism.	(Tsai et al., 2002; Guzman et al., 2003; Grover and Luthra, 2013; Acharya et al., 2017; Dror et al., 2017; Liccardo et al., 2019; Turk Wensveen et al., 2021)
Role of oral bacteria	Periodontitis is caused by pathogenic oral bacteria, which may enter the bloodstream.	T2DM can increase the risk of periodontal disease due to immune dysfunction.	Oral bacteria may contribute to systemic inflammation, increasing COVID-19 severity.	(Nussbaum and Shapira, 2011; Ling et al., 2015; Sukumar and Tadepalli, 2021; Yin et al., 2021)
Risk of complications	Increased risk of systemic diseases.	Patients with T2DM are at higher risk for severe COVID-19 outcomes.	COVID-19 can worsen outcomes in patients with existing comorbidities, including periodontitis and T2DM.	(Acharya et al., 2017; Moreno Caicedo et al., 2018; Shenoy et al., 2020; You et al., 2020; Brock et al., 2022)
Risk of co-infection	Periodontitis can increase the risk of respiratory infections.	T2DM patients are at higher risk of infections, including periodontitis and respiratory infections.	COVID-19 can increase susceptibility to secondary bacterial infections, including oral infections.	(Dhir et al., 2018; Anand et al., 2022; Brock et al., 2022; Kalsi et al., 2022; Sánchez Sánchez et al., 2022)

*Clinicopathological axis*. Regarding this axis, studies have reported that patients who suffer from T2DM are more susceptible to developing periodontitis, even in a more severe course of this disease. In turn, patients with poorly controlled T2DM have a higher prevalence of periodontitis with a more severe course (Tsai et al., 2002; Susanto et al., 2011; Shamala et al., 2017; Alasqah et al., 2018; Trentin et al., 2018; Monod Nuñez et al., 2022), evidencing a bidirectional relationship between both pathologies (Wu et al., 2020). In turn, poor control of T2DM is associated with high morbidity and mortality by COVID-19, increasing the risk of death, admission to the intensive care unit, and receiving mechanical ventilation (Shenoy et al., 2020; You et al., 2020; Turk Wensveen et al., 2021). Likewise, it has been reported that patients with COVD-19 show a more severe course of periodontitis, and that this, in turn, is associated with complications during COVID-19 disease, including death, admission to the care unit intensive care, need for assisted ventilation and pneumonia (Marouf et al., 2021; Anand et al., 2022; Brock et al., 2022; Gupta et al., 2022; Kalsi et al., 2022).

*Axis associated with glycemia.* One of the main characteristics of T2DM is the lack of control of blood glucose, since, if the disease is not controlled, patients who suffer from it, present hyperglycemia. In this context, hyperglycemia triggers many negative effects on the health of patients, including making them more prone to the development of comorbidities with other diseases. On the one hand, hyperglycemia in patients with T2DM favors inflammatory mechanisms that, in turn, can induce insulin resistance (Acharya et al., 2017), decreased endothelial function (Kumar et al., 2020), and an increase in PMN (Herrmann et al., 2020). These factors influence in periodontitis, enhancing the destruction of periodontal tissues, due to the exacerbation of the inflammatory response, generating a more serious course of the disease (Arreguin-Cano et al., 2019). In this context, these conditions favor the infectious capacity of SARS-CoV-2 (Sukumar and Tadepalli, 2021). This is because hyperglycemia is a key factor in the development of T2DM. Therefore, high glucose levels can make patients with T2DM more susceptible to COVID-19, as hyperglycemia can affect the production of enzymes that help the SARS-CoV-2 infect and multiply in the body (Michaels et al., 2024). Furthermore, hyperglycemia can worsen the inflammatory response of the body to the virus. Furthermore, periodontal-pathogenic bacteria, if aspirated into the lungs, induce the overexpression of ACE-2 in the alveoli (Campisi et al., 2021; Sukumar and Tadepalli, 2021), which favors lung inflammation and exacerbated production of proinflammatory cytokines, generating a cytokine storm that induces the destruction of the resident tissue (Campisi et al., 2021; Brock et al., 2022). This phenomenon is also closely related to T2DM, since hyperglycemia further enhances the cytokine storm at the lung level, thus increasing the inflammatory response, due to a deterioration of the immune system (Yin et al., 2021), caused by T2DM, which favors the severity of COVID-19 disease (Turk Wensveen et al., 2021).

*The immune axis associated with inflammation*. The integration of this axis is even more complex, due to the interconnected pathways between periodontitis, T2DM and COVID-19 disease. However, the common denominator within the axis is inflammation. Periodontitis is caused, mainly, by the inflammatory response induced by periodontal-pathogenic bacteria residing in dental plaque (Kinane, 2001). The chronicity of this inflammatory response is characterized by an increase in proinflammatory cytokines, such as TNF-α, IL-1β and IL-6, and immune system cell populations (Acharya et al., 2017). Particularly, the aberrant production of TNF-α, on the one hand, generates decreased vascular function (Kumar et al., 2020). On the other hand, it induces the increase and survival of PMN in the periodontal tissue (Manosudprasit et al., 2017; Herrmann et al., 2020), which in turn produces MMP-2, which leads to the destruction of periodontal tissue (Arreguin-Cano et al., 2019). Likewise, TNF-α modulates the expression of endocan, a proteoglycan that acts as a pro-inflammatory mediator, which is associated with the most severe course of the disease (Kumar et al., 2020). Regarding IL-1β, this proinflammatory cytokine is associated with the activation of the inflammasome (they are over expression of NLRP3), amplifying the inflammatory response and therefore the destruction of gingival tissue (Huang et al., 2015). The intersection between periodontitis, T2DM and COVID-19 disease (Sukumar and Tadepalli, 2021; Brock et al., 2022), occurs when during diabetes mellitus, there is an increase in blood glucose levels (hyperglycemia) and together with the viral infection, an exacerbated inflammatory response is triggered, increasing the production of TNF-α. IL-1β, IL-6, endocan, NLRP3 inflammasome and an increase in the PMN population, amplifying their effects and leading to a more severe course of comorbidity between these three pathologies (Huang et al., 2015; Acharya et al., 2017; Kumar et al., 2020; Qi et al., 2023). In turn, during COVID-19 disease, periodontitis facilitates the passage of periodontal-pathogenic bacteria, invading the lung, which increase the expression of ACE-2, favoring SARS-CoV-2 infection (Campisi et al., 2021; Sukumar and Tadepalli, 2021); which in turn produces a strong inflammatory response, also characterized by the aberrant production of proinflammatory cytokines (TNF-α, IL-1β and IL-6) (Torres-Tamayo et al., 2020; Silvestre and Márquez-Arrico, 2022; Qi et al., 2023), and the activation of alveolar macrophages, which leads to a cytokine storm (Yin et al., 2021), which ultimately induces tissue destruction at the lung level (Campisi et al., 2021), generating respiratory failure (Campisi et al., 2021; Brock et al., 2022). However, this cytokine storm manages to reach the systemic circulation, which reaches the periodontal tissues, also favoring their destruction (Ariana Dalys and Fabricio Miltom, 2023)

## 6 Conclusion

Currently, scientific evidence that jointly analyzes the relationship between periodontitis, T2DM and the risk of developing COVID-19 remains limited. However, based on a systematic review of available studies, it is possible to propose a well-founded hypothesis that suggests the existence of three key axes linking these conditions. The three diseases share similar clinical characteristics, such as chronic inflammation, progressive tissue deterioration, and dysregulated immune responses, suggesting common pathological mechanisms that could enhance their interaction. Both T2DM and periodontitis are closely related to glycemic control. Persistent hyperglycemia creates an environment conducive to the development of infections and exacerbates inflammatory processes, which can increase susceptibility to complications in the event of SARS-CoV-2 infection. Therefore, these three diseases involve an alteration of the immune system, characterized by an excessive or dysregulated inflammatory response. This condition could explain why patients with T2DM and periodontitis are at greater risk of developing severe forms of COVID-19, as they generate a more intense and damaging immune response to the virus. Taken together, this interrelationship suggests that comorbidity between periodontitis and T2DM not only increases vulnerability to COVID-19 infection but can also lead to a more severe clinical course of the disease.
